# Maternal factors and child health conditions at birth associated with preterm deaths in a tertiary health facility in Ghana: A retrospective analysis

**DOI:** 10.3389/fpubh.2023.1108744

**Published:** 2023-02-09

**Authors:** Seth Kwaku Afagbedzi, Yakubu Alhassan, Deda Ogum Alangea, Henrietta Taylor

**Affiliations:** ^1^Department of Biostatistics, School of Public Health, University of Ghana, Accra, Ghana; ^2^Department of Population, Family and Reproductive Health, School of Public Health, University of Ghana, Accra, Ghana; ^3^Paediatric Department, 37 Military Hospital, Accra, Ghana

**Keywords:** preterm birth, maternal factors, child health conditions at birth, preterm death, newborn, Ghana

## Abstract

**Background:**

Preterm birth continues to be a leading cause of death for children under the age of 5 globally. This issue carries significant economic, psychological, and social costs for the families affected. Therefore, it is important to utilize available data to further research and understand the risk factors for preterm death.

**Objective:**

The objective of this study was to determine maternal and infant complications that influence preterm deaths in a tertiary health facility in Ghana.

**Methods:**

A retrospective analysis of data on preterm newborns was conducted at the neonatal intensive care unit of Korle Bu Teaching Hospital (KBTH NICU) in Ghana, covering the period January 2017 to May 2019. Pearson's Chi-square test of association was used to identify factors that were significantly associated with preterm death after admission at the NICU. The Poisson regression model was used to determine the risk factors of preterm death before discharge after admission to the NICU.

**Results:**

Of the 1,203 preterm newborns admitted to the NICU in about two and half years, 355 (29.5%) died before discharge, 7.0% (*n* = 84) had normal birth weight (>2.5 kg), 3.3% (*n* = 40) had congenital anomalies and 30.5% (*n* = 367) were born between 34 and 37 gestational week. All 29 preterm newborns between the 18–25 gestational week died. None of the maternal conditions were significant risk factors of preterm death in the multivariable analysis. The risk of death at discharge was higher among preterm newborns with complications including hemorrhagic/hematological disorders of fetus (aRRR: 4.20, 95% CI: [1.70–10.35], *p* = 0.002), fetus/newborn infections (aRRR: 3.04, 95% CI: [1.02–9.04], *p* = 0.046), respiratory disorders (aRRR: 13.08, 95% CI: [5.50–31.10], *p* < 0.001), fetal growth disorders/restrictons (aRRR: 8.62, 95% CI: [3.64–20.43], *p* < 0.001) and other complications (aRRR: 14.57, 95% CI: [5.93–35.77], *p* < 0.001).

**Conclusion:**

This study demonstrate that maternal factors are not significant risk factors of preterm deaths. Gestational age, birth weight, presence of complications and congenital anomalies at birth are significantly associated with preterm deaths. Interventions should focus more on child health conditions at birth to reduce the death of preterm newborns.

## Introduction

Preterm birth, since 2016, has been identified as the leading cause of under 5 mortality globally (1, 2). Previously, pneumonia was the leading cause of death with more than half of deaths before age 5 years, but the main cause of death has now shifted to prematurity (3). This shift in epidemiology of infant mortality from pneumonia to preterm birth, should increase concern for and investment into addressing prematurity (1). As at 2010, more than 60% of preterm births occurred in south Asia and Sub-Sahara Africa (SSA) (4), which increased to 81.1% in 2016 (5). Moreover, the highest rates of preterm deaths have been recorded in West Africa at ~16 per 1,000 live births (1).

Prematurity not only contributes directly to neonatal deaths globally but also increases the risk of death from other causes (6). These causes are multifactorial, including infant biological and medical conditions, such as birthweight, gestational age, (7–10), congenital anomalies, birth complications (2, 3), respiratory problems, jaundice, sepsis, necrotizing enterocolitis, intrapartum asphyxia, seizures, bleeding disorders among others (11, 12). Preterm babies are more affected by these factors because they are physiologically and metabolically less mature than term infants (13). Maternal factors such as age, hypertensive disorders, height, pre-gestational diabetes and pre-eclampsia also increase the risk of preterm deaths (12, 14, 15). Preterm births are not only associated with deaths but also with high economic, psychological, and social costs incurred by affected families (16, 17).

Risk factors of preterm births, which also contribute to a large proportion of preterm deaths (18), have been less explored in low-to-middle-income (LMIC) settings compared to high income countries (HIC). Thus, they are poorly understood and may be totally different in the former (1). It has been projected that if current trends continue in preterm births among other causes of death, SSA will record about 60% of the under-five deaths even in 2030 (3). Prematurity has also been found as one of the top five causes of neonatal deaths in Ghana (19, 20). As at 2010, the estimated preterm birth rate for the country was 14.5 per 100 live births, which reduced to 12 per 100 live births in 2019 with over 8,000 deaths per year (21, 22). It is obvious Ghana has fallen short of its target to reduce the NMR to 21 per 1,000 live births in 2018. This is indicated in an estimated rate of 24 deaths per 1,000 live births in the Ghana National Newborn Health Strategy and Action Plan for 2014–2018 (20). Addressing the national burden of preterm-related neonatal and child mortality is crucial to achieve the SDG 3.2. This study seeks to explore maternal and infant factors influencing preterm births and deaths in the nation's largest tertiary referral hospital. The current analysis offers the opportunity to utilise routine data which is readily available to answer pertinent questions on preterm birth and death with relatively less resources.

## Methods

### Data source

A retrospective analysis of secondary data on preterm infants was conducted at the neonatal intensive care unit of the Korle Bu Teaching Hospital (KBTH NICU) in Ghana. Data extraction forms were used to collect data from folders from the pediatric department, the admission and discharge book at the nurse's desk, including copies of death certificates covering the period January 2017 to May 2019. All medical records of preterm infants pronounced dead on arrival at KBTH NICU, and those on preterm infants with incomplete data were excluded. Korle Bu Teaching Hospital NICU is a tertiary referral centre that is equipped with 20 incubators, 20 cots, and 10 radiant warmers. The unit manages ~2,500 newborns annually with about 10 new admissions each day. Preterm babies account for 50–60% of all new admissions. About 80% of the newborns admitted at the unit are referred from the KBTH labor wards and obstetric theatre. The rest are referred from peripheral hospitals. Data extraction span over 5 weeks with the support of five research assistants.

### Outcome measure

The primary dependent variable for this study was the discharge outcome at NICU among preterm births, that is whether the preterm infant was dead or alive before discharge from NICU. Preterm infant deaths before discharge were confirmed from the duplicates of death certificates kept at NICU.

### Exposure variables

Exposure variables were subdivided into maternal, delivery-related, and infant factors. Maternal factors included sociodemographic characteristics of the preterm infant's mother such as age, occupation, marital status, educational status, religion, parity, and HIV status of mother as well as maternal medical conditions. The place of delivery (whether at KBTH or other facilities), mode of delivery (either by caesarian section or vaginal delivery), and whether any resuscitation was done in an attempt to stabilize the newly delivered preterm infant, constituted delivery-related factors. Infant factors including sex of infant, gestational age in weeks at birth, birth weight, as well as the presence or absence of congenital anomalies and complications were also assessed.

Birth complications in the preterm newborns were categorized using the WHO's International Classification of Diseases (ICD-11) for Mortality and Morbidity Statistics (Version: 04 / 2019) (23). The categories included hemorrhagic/hematological disorders of the fetus, fetus/newborn infections [this is based on clinical evaluation and lab tests (cultures, CRP)], respiratory disorders, fetal growth disorders, and others (digestive system disorders, transitory endocrine/metabolic disorders, disturbances of temperature regulation, neurological disorders and, genitourinary system disorders). Gestational age at delivery were categorized as extreme preterm (< 28 weeks), very preterm (28 to < 32 weeks), moderate preterm (32 to < 34 weeks), and late preterm (34 to < 37 weeks). Also, the infant's birth weight in kilograms (kg) was categorized as normal (>2.5 kg), low birth weight (< 2.5–1.5 kg), very low birth weight (< 1.5–1.0 kg), and extremely low birth weight (< 1.0 kg).

### Data analysis

The data extracted from the medical records were cleaned, coded, and entered into a Microsoft excel 2016 spreadsheet and exported into Stata IC version 16 (Stata Corp, College Station, TX, US) for final analysis. Descriptive statistics including frequencies, proportions, mean, and standard deviation were were used to describe the maternal and child characteristics by gestational age at bith. The line plot was also used to describe the preterm outcome after admission at NICU by the completed gestational age at birth in weeks.

Pearson's Chi-square test of association was used to identify factors that were significantly associated with preterm outcomes after admission at the NICU. The Poisson regression model was used to determine the risk factors of preterm death before discharge after admission to the NICU. The variance inflation factor (VIF) was used to assess multicollinearity. The final multivariable model had a average VIF of 2.66 (range: 1.07–7.08) below the threshold of 10. The deviance and Pearson's goodness of fit test were both non-significant with *p-*values of 1.000 indicating the model is appropriate. The statistical significance for the study was set at a *p-*value of 0.05.

### Missingness and completeness of data used for analysis

**Figure 1:**
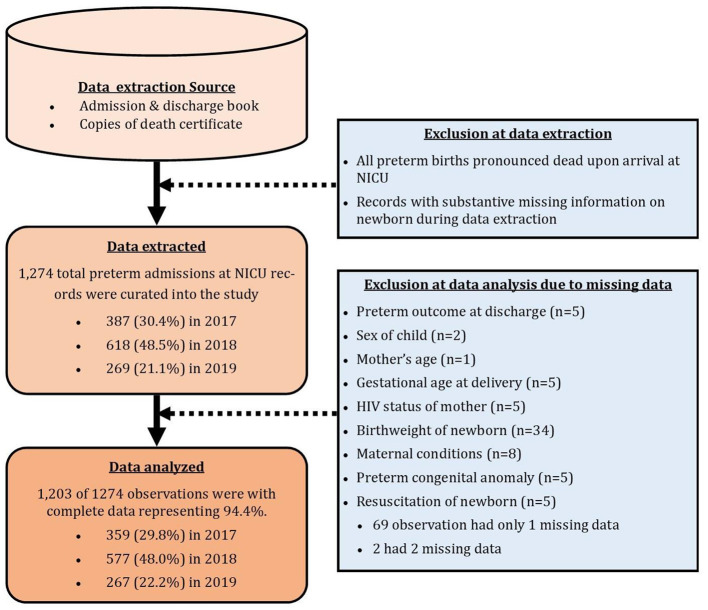
Flow chart of data extraction, showing missingness and final data used for analysis.

Medical records of a total of 1,274 preterm newborns admitted into the NICU of Korle-Bu Teaching Hospital during the years 2017 (*n* = 387, 30.4%), 2018 (*n* = 618, 48.5%), and 2019 (*n* = 269, 21.1%) were reviewed. Due to missingness on some very important variables in the study, a total of 1,203 observations with complete information were used for the analysis of this study, 359 (29.8%) from 2017, 577 (48.0%) from 2018, and 267 (22.2%) from 2019 records (Figure 1).

## Results

### Descriptive characteristics of study participants

The average age of the 1,203 mothers whose newborns were admitted to the NICU was 30.0 (±6.4) years with most (*n* = 568, 47.2%) of them in the age range 30–39 years. A few 3.9% (*n* = 47) had no formal education whilst 18.7% (*n* = 225) of them had a tertiary level of education. One thousand and six (83.6%) were married and 54.0% (*n* = 650) of them had given birth to only one child. Fifteen (1.25%) of the mothers were HIV positive. The majority (*n* = 1,057, 87.9%) of the preterm newborns were delivered at Korle-Bu Teaching Hospital, with over a third (*n* = 323, 37.3%) of the births through cesarean section. About half (*n* = 583, 48.5%) of the mothers had no maternal medical conditions. Among the 38 with maternal infectious diseases, hepatitis B was the common with 17, followed by syphilis with 12, urinary tract infection with 5 and 4 with candidiasis. About 50.5% of the preterm infants were males. Of the 1,207 preterm newborns admitted to the NICU, 15.9% (*n* = 192) of them had no complications and 42.8% of them required resuscitation. Less than a tenth (*n* = 84, 7.0%) were born with normal birth weight (>2.5 kg), 3.3% had congenital anomalies and about a third (*n* = 367, 30.5%) were born at 34 to 37 weeks (Table 1).

**Table 1:** Descriptive characteristics of child, maternal and delivery-related factors by gestational age at birth.

		**Gestational week at birth of preterm newborns**				**Chi-square (DF)**	***P-*value**
	**Total**	**Late** **(34 to**<**37)**	**Moderate** **(32 to**<**34)**	**Very** **(28 to**<**32)**	**Extreme** **(**<**28)**		
	***N** =* **1,203**	***N** =* **367**	***N** =* **346**	***N** =* **320**	***N** =* **170**		
**Characteristics**	***n*** **(%)**	***n*** **(%)**	***n*** **(%)**	***n*** **(%)**	***n*** **(%)**		
**Maternal**							
**Mother's age in years: Mean [±SD]**	30.0 [±6.3]	30.1 [±6.4]	30.4 [±6.3]	29.8 [±6.3]	29.4 [±6.5]		
**Mother's age in years**						12.47 (9)	0.190
< 20	66 (5.5)	14 (3.8)	16 (4.6)	22 (6.9)	14 (8.2)		
20–29	490 (40.7)	155 (42.2)	138 (39.9)	124 (38.8)	73 (42.9)		
30–39	568 (47.2)	176 (48.0)	164 (47.4)	159 (49.7)	69 (40.6)		
40–49	79 (6.6)	22 (6.0)	28 (8.1)	15 (4.7)	14 (8.2)		
**Mother's education**						18.20 (12)	0.110
None	47 (3.9)	20 (5.4)	11 (3.2)	11 (3.4)	5 (2.9)		
Primary	247 (20.5)	65 (17.7)	83 (24.0)	57 (17.8)	42 (24.7)		
Junior high school	469 (39.0)	154 (42.0)	137 (39.6)	126 (39.4)	52 (30.6)		
Senior high school	215 (17.9)	59 (16.1)	61 (17.6)	63 (19.7)	32 (18.8)		
Tertiary	225 (18.7)	69 (18.8)	54 (15.6)	63 (19.7)	39 (22.9)		
**Marital status**						10.08 (6)	0.120
Single	84 (7.0)	19 (5.2)	24 (6.9)	22 (6.9)	19 (11.2)		
Co-habiting	113 (9.4)	33 (9.0)	27 (7.8)	32 (10.0)	21 (12.4)		
Married	1,006 (83.6)	315 (85.8)	295 (85.3)	266 (83.1)	130 (76.5)		
**Religion**						6.57 (3)	0.087
Non-Christians	138 (11.5)	55 (15.0)	36 (10.4)	31 (9.7)	16 (9.4)		
Christians	1,065 (88.5)	312 (85.0)	310 (89.6)	289 (90.3)	154 (90.6)		
**Parity**						4.50 (6)	0.610
One	650 (54.0)	191 (52.0)	184 (53.2)	174 (54.4)	101 (59.4)		
Two	272 (22.6)	88 (24.0)	80 (23.1)	66 (20.6)	38 (22.4)		
Three or more	281 (23.4)	88 (24.0)	82 (23.7)	80 (25.0)	31 (18.2)		
**Mother's occupation**						19.78 (6)	0.003
Unemployed	591 (49.1)	194 (52.9)	169 (48.8)	159 (49.7)	69 (40.6)		
Informal sector	380 (31.6)	103 (28.1)	128 (37.0)	95 (29.7)	54 (31.8)		
Formal sector	232 (19.3)	70 (19.1)	49 (14.2)	66 (20.6)	47 (27.6)		
**HIV status**						7.22 (3)	0.065
Negative	1,188 (98.8)	358 (97.5)	342 (98.8)	319 (99.7)	169 (99.4)		
Positive	15 (1.2)	9 (2.5)	4 (1.2)	1 (0.3)	1 (0.6)		
**Delivery related**							
**Place of delivery**						6.92 (3)	0.074
Korle-Bu	1,057 (87.9)	324 (88.3)	314 (90.8)	278 (86.9)	141 (82.9)		
Other	146 (12.1)	43 (11.7)	32 (9.2)	42 (13.1)	29 (17.1)		
**Mode delivery**						55.87 (3)	< 0.001
Caesarean section	656 (54.5)	215 (58.6)	199 (57.5)	194 (60.6)	48 (28.2)		
Vaginal delivery	547 (45.5)	152 (41.4)	147 (42.5)	126 (39.4)	122 (71.8)		
**Maternal conditions**						48.62 (18)	< 0.001
None	583 (48.5)	168 (45.8)	167 (48.3)	138 (43.1)	110 (64.7)		
Disorders in pregnancy	371 (30.8)	110 (30.0)	111 (32.1)	123 (38.4)	27 (15.9)		
Obstetric hemorrhage	19 (1.6)	6 (1.6)	3 (0.9)	4 (1.3)	6 (3.5)		
Maternal disorders	49 (4.1)	18 (4.9)	17 (4.9)	9 (2.8)	5 (2.9)		
Fetus/amniotic/delivery related conditions	126 (10.5)	41 (11.2)	34 (9.8)	36 (11.3)	15 (8.8)		
Maternal infectious diseases (Hep. B, syphilis, etc.)	38 (3.2)	18 (4.9)	10 (2.9)	4 (1.3)	6 (3.5)		
Unspecified conditions	17 (1.4)	6 (1.6)	4 (1.2)	6 (1.9)	1 (0.6)		
**Child**							
**Sex**						3.70 (3)	0.300
Female	608 (50.5)	172 (46.9)	174 (50.3)	172 (53.8)	90 (52.9)		
Male	595 (49.5)	195 (53.1)	172 (49.7)	148 (46.3)	80 (47.1)		
**Complications on admission**						111.12 (15)	< 0.001
None	192 (16.0)	78 (21.3)	61 (17.6)	44 (13.8)	9 (5.3)		
Hemorrhagic/hematological disorders of fetus	250 (20.8)	89 (24.3)	92 (26.6)	56 (17.5)	13 (7.6)		
Fetus/newborn infections	83 (6.9)	33 (9.0)	25 (7.2)	17 (5.3)	8 (4.7)		
Respiratory disorders	349 (29.0)	72 (19.6)	78 (22.5)	107 (33.4)	92 (54.1)		
Fetal growth disorders/restrictions	285 (23.7)	76 (20.7)	83 (24.0)	83 (25.9)	43 (25.3)		
Others	44 (3.7)	19 (5.2)	7 (2.0)	13 (4.1)	5 (2.9)		
**Resuscitation**						16.89 (3)	< 0.001
No	688 (57.2)	221 (60.2)	216 (62.4)	175 (54.7)	76 (44.7)		
Yes	515 (42.8)	146 (39.8)	130 (37.6)	145 (45.3)	94 (55.3)		
**Birth weight**						616.34 (9)	< 0.001
Normal (≥2.5 kg)	84 (7.0)	62 (16.9)	17 (4.9)	1 (0.3)	4 (2.4)		
Low birth weight (1.5 to < 2.5 kg)	621 (51.6)	255 (69.5)	243 (70.2)	114 (35.6)	9 (5.3)		
Very low birth weight (1.0 to < 1.5 kg)	358 (29.8)	47 (12.8)	83 (24.0)	160 (50.0)	68 (40.0)		
Extremely low birth weight (< 1.0 kg)	140 (11.6)	3 (0.8)	3 (0.9)	45 (14.1)	89 (52.4)		
**Congenital anomalies**						9.48 (3)	< 0.001
No	1,163 (96.7)	349 (95.1)	331 (95.7)	315 (98.4)	168 (98.8)		
Yes	40 (3.3)	18 (4.9)	15 (4.3)	5 (1.6)	2 (1.2)		

N (%), Frequency (Column percentage); DF, Degree of freedom; SD, Standard deviation.

Table 1 also shows the descriptive characteristics of the participants by the gestational age at birth. The gestational age at birth of the newborns varied significantly across some of the characteristics including mode of delivery, maternal condition, complication of child at NICU admission, resusicitation, birthweight and presence of congenital anormaly (Table 1).

### Outcome of care among preterm newborns admitted at the NICU

Of the 1,203 preterm newborns admitted at the NICU, 29.5% (*n* = 355) died before discharge. Marital status [χ^2^(2) = 7.2, *p-*value = 0.028] was significantly associated with outcome at discharge with higher mortality among single mothers (40.5%) compared to co-habiting (23.0%) and married mothers (29.3%). Also, place of delivery [χ^2^(1) = 10.72, *p-*value = 0.001] was a significant factor with higher mortality among mothers who delivered elsewhere (41.1%) compared to those who delivered in Korle-Bu (27.9%). Mode of delivery [χ^2^(1) = 3.9, *p-*value = 0.048] was associated with outcome at discharge with higher mortality among vaginal delivery (32.4%) compared to caesarean section delivery (27.1%). Death at discharge from NICU was also higher among preterm newborns who were resuscitated (38.8%) compared to the non-resuscitated (22.5%) [χ^2^(1) = 37.7, *p-*value < 0.001]. Mortality at discharge from NICU was higher among preterm births newborns with lower birthweight [χ^2^(3) = 314.2, *p-*value < 0.001]. Also, mortality at discharge from NICU was higher among newborns with congenital anomaly [χ^2^(1) = 10.5, *p-*value = 0.001]. Gestational weeks at birth was also associated with moratlity at NICU discharge [χ^2^(3) = 245.6, *p-*value < 0.001] were the factors that significantly influenced the outcome of preterm infants at the KBTH NICU (Table 2).

**Table 2:** Prevalence and association between death before discharge and demographic characteristics among preterm newborns admitted at the NICU.

		**Outcone at discharge**			
	**Total**	**Alive**	**Died**	**Chi-square (DF)**	***P-*** **value**
**Characteristics**		***n*** **(%)**	***n*** **(%)**		
**Overall**	**1,203**	**848 (70.5)**	**355 (29.5)**		
**MATERNAL**					
**Mother's age in years**				1.37 (3)	0.713
< 20	66	47 (71.2)	19 (28.8)		
20-29	490	337 (68.8)	153 (31.2)		
30-39	568	409 (72.0)	159 (28.0)		
40-49	79	55 (69.6)	24 (30.4)		
**Mother's education**				7.47 (4)	0.113
None	47	40 (85.1)	7 (14.9)		
Primary	247	176 (71.3)	71 (28.7)		
Junior high school	469	336 (71.6)	133 (28.4)		
Senior high school	215	146 (67.9)	69 (32.1)		
Tertiary	225	150 (66.7)	75 (33.3)		
**Marital status**				7.17 (2)	0.028
Single	84	50 (59.5)	34 (40.5)		
Co-habiting	113	87 (77.0)	26 (23.0)		
Married	1,006	711 (70.7)	295 (29.3)		
**Religion**				0.55 (1)	0.460
Non-Christians	138	101 (73.2)	37 (26.8)		
Christians	1,065	747 (70.1)	318 (29.9)		
**Parity**				2.73 (2)	0.255
One	650	461 (70.9)	189 (29.1)		
Two	272	199 (73.2)	73 (26.8)		
Three or more	281	188 (66.9)	93 (33.1)		
**Mother's occupation**				4.72 (2)	0.094
Unemployed	591	424 (71.7)	167 (28.3)		
Informal sector	380	274 (72.1)	106 (27.9)		
Formal sector	232	150 (64.7)	82 (35.3)		
**HIV status**				0.66 (1)	0.416
Negative	1,188	836 (70.4)	352 (29.6)		
Positive	15	12 (80.0)	3 (20.0)		
**Delivery-related**					
**Place of delivery**				10.72 (1)	0.001
Korle-Bu	1,057	762 (72.1)	295 (27.9)		
Other	146	86 (58.9)	60 (41.1)		
**Mode delivery**				3.91 (1)	0.048
Caesarean section	656	478 (72.9)	178 (27.1)		
Vaginal delivery	547	370 (67.6)	177 (32.4)		
**Maternal medical conditions**				12.41 (6)	0.053
None	583	403 (69.1)	180 (30.9)		
Disorders in pregnancy	371	258 (69.5)	113 (30.5)		
Obstetric hemorrhage	19	11 (57.9)	8 (42.1)		
Maternal disorders	49	41 (83.7)	8 (16.3)		
Fetus, amniotic and delivery related conditions	126	98 (77.8)	28 (22.2)		
Maternal infectious diseases (Hep. B, syphilis, etc.)	38	23 (60.5)	15 (39.5)		
Unspecified conditions	17	14 (82.4)	3 (17.6)		
**Child**					
**Sex of child**				0.01 (1)	0.941
Female	608	428 (70.4)	180 (29.6)		
Male	595	420 (70.6)	175 (29.4)		
**Complications on admission**				272.08 (1)	< 0.001
None	192	187 (97.4)	5 (2.6)		
Hemorrhagic/hematological disorders of fetus	250	222 (88.8)	28 (11.2)		
Fetus/newborn infections	83	76 (91.6)	7 (8.4)		
Respiratory disorders	349	146 (41.8)	203 (58.2)		
Fetal growth disorders/restrictions	285	195 (68.4)	90 (31.6)		
Others	44	22 (50.0)	22 (50.0)		
**Resuscitation**				37.65 (1)	< 0.001
No	688	533 (77.5)	155 (22.5)		
Yes	515	315 (61.2)	200 (38.8)		
**Birth weight**				314.20 (3)	< 0.001
Normal (≥2.5 kg)	84	76 (90.5)	8 (9.5)		
Low birth weight (1.5 to < 2.5 kg)	621	529 (85.2)	92 (14.8)		
Very low birth weight (1.0 to < 1.5 kg)	358	225 (62.8)	133 (37.2)		
Extremely low birth weight (< 1.0 kg)	140	18 (12.9)	122 (87.1)		
**Completed gestational week at birth**				245.56 (3)	< 0.001
Late (34 to < 37 weeks)	367	305 (83.1)	62 (16.9)		
Moderate preterm (32 to < 34 weeks)	346	297 (85.8)	49 (14.2)		
Very preterm (28 to < 32 weeks)	320	204 (63.7)	116 (36.3)		
Extreme preterm (< 28 weeks)	170	42 (24.7)	128 (75.3)		
**Congenital anomalies**				10.51 (1)	0.001
No	1,163	829 (71.3)	334 (28.7)		
Yes	40	19 (47.5)	21 (52.5)		

N (%), Frequency (Row percentage); DF, Degree of freedom.

### Completed gestational age in weeks at birth and Preterm outcome after admission at NICU

**Figure 2:**
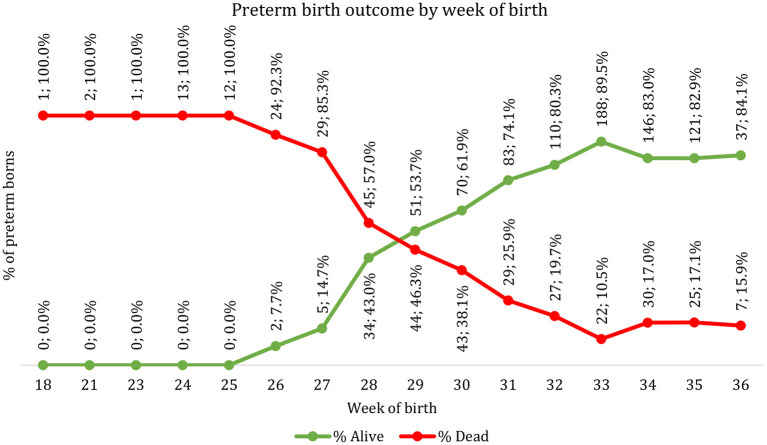
Preterm outcome by the week of birth.

Figure 2 shows the oucomes dead and alive outcomes before the discharge of preterm newborns admitted to the NICUby their completed gestational age in weeks at birth. All 29 preterm newborns with gestational age < 26 weeks died. Only 2 (7.7%) of the 26 newborns during the 26th gestational week survived whilst 43.0% (*n* = 34/79) of the newborns during the 28th gestational week survived. On the other hand, mortality were 17.1% and 15.9% among the newborns on the 35 and 36th week, respectively. The proportion of preterm newborns surviving at each gestational age increased steeply between 27 and 28th gestational weeks (from 14.7 to 43%) then increased steadily to 32nd gestational weeks before levelling from 34 to 36th gestational weeks. Proportionately more preterm before the 29th gestational weeks died than survive (57% of preterm die at 28 weeks). Half of the preterm newborns born between the 29 and 30th gestational week survived.

### Risk factors of death before discharge among preterm newborns admitted at the NICU

Table 3 shows the unadjusted and adjusted Poisson regression model of risk factors of death outcome among NICU admitted preterm newborns. From the adjusted Poisson model, maternal conditions were not significant risk factors of preterm death. The risk of death at discharge was significantly higher among new borns with with complications including hemorrhagic/hematological disorders of the fetus (aRRR: 4.20, 95% CI: [1.70–10.35], *p* = 0.002), fetus/newborn infections (aRRR: 3.04, 95% CI: [1.02–9.04], *p* = 0.046), respiratory disorders (aRRR: 13.08, 95% CI: [5.50–31.10], *p* < 0.001), fetal growth disorders (aRRR: 8.62, 95% CI: [3.64–20.43], *p* < 0.001) and other complications (aRRR: 14.57, 95% CI: [5.93–35.77], *p* < 0.001).

**Table 3:** Risk factors of death before discharge among preterm newborns admitted at the NICU.

**Characteristics**	**Poisson regression model of risk factor of death at discharge**			
	**Unadjusted model**		**Adjusted model**	
	**uRRR [95% CI]**	***P-*** **value**	**aRRR [95% CI]**	***P-*** **value**
**MATERNAL**				
**Mother's education**				
None	1.00 [reference]		1.00 [reference]	
Primary	1.93 [0.89–4.20]	0.097	1.20 [0.67, 2.15]	0.532
Junior high school	1.90 [0.89–4.07]	0.097	1.31 [0.74, 2.32]	0.362
Senior high school	2.15 [0.99–4.69]	0.053	1.25 [0.69, 2.27]	0.452
Tertiary	2.24 [1.03–4.86]	0.042	1.17 [0.61, 2.26]	0.635
**Marital status**				
Single	1.38 [0.97, 1.97]	0.075	1.25 [0.99, 1.58]	0.066
Co-habiting	0.78 [0.53, 1.17]	0.236	0.85 [0.64, 1.12]	0.247
Married	1.00 [reference]		1.00 [reference]	
**Religion**				
Non-Christians	1.00 [reference]		1.00 [reference]	
Christians	1.11 [0.79–1.57]	0.535	0.99 [0.79, 1.25]	0.941
**Parity**	1.05 [0.93–1.19]	0.407	1.11 [1.01, 1.21]	0.029
**Mother's occupation**				
Unemployed	1.00 [reference]		1.00 [reference]	
Informal sector	0.99 [0.77–1.26]	0.917	0.98 [0.82, 1.16]	0.810
Formal sector	1.25 [0.96–1.63]	0.097	1.13 [0.82, 1.57]	0.455
**HIV status**				
Negative	1.00 [reference]		1.00 [reference]	
Positive	0.68 [0.22–2.10]	0.498	1.11 [0.43, 2.82]	0.833
**Delivery-related**				
**Place of delivery**				
Korle-Bu	1.00 [reference]		1.00 [reference]	
Other	1.47 [1.12–1.94]	0.006	1.14 [0.95, 1.38]	0.166
**Mode delivery**				
Caesarean section	1.00 [reference]		1.00 [reference]	
Vaginal delivery	1.19 [0.97–1.47]	0.097	1.00 [0.83, 1.21]	0.997
**Maternal medical conditions**				
None	1.00 [reference]		1.00 [reference]	
Disorders in pregnancy	0.99 [0.78–1.25]	0.910	1.21 [0.99, 1.48]	0.058
Obstetric hemorrhage	1.36 [0.67–2.77]	0.391	1.17 [0.76, 1.79]	0.479
Maternal disorders	0.53 [0.26–1.07]	0.078	0.87 [0.49, 1.54]	0.629
Fetus, amniotic and delivery related conditions	0.72 [0.48–1.07]	0.106	0.98 [0.74, 1.31]	0.917
Maternal infectious diseases (Hep. B, syphilis, etc.)	1.28 [0.76–2.16]	0.361	1.36 [0.89, 2.06]	0.155
Unspecified conditions	0.57 [0.18–1.79]	0.337	0.83 [0.38, 1.84]	0.655
**Child**				
**Sex of child**				
Female	1.00 [reference]		1.00 [reference]	
Male	0.99 [0.81–1.22]	0.951	1.11 [0.97, 1.28]	0.137
**Complications on admission**				
None	1.00 [reference]		1.00 [reference]	
Hemorrhagic/hematological disorders of fetus	4.30 [1.66–11.14]	0.003	4.20 [1.70, 10.35]	0.002
Fetus/newborn infections	3.24 [1.03–10.20]	0.045	3.04 [1.02, 9.04]	0.046
Respiratory disorders	22.34 [9.20–54.24]	< 0.001	13.08 [5.50, 31.10]	< 0.001
Fetal growth disorders/restrictions	12.13 [4.93–29.84]	< 0.001	8.62 [3.64, 20.43]	< 0.001
Others	19.20 [7.27–50.70]	< 0.001	14.57 [5.93, 35.77]	< 0.001
**Resuscitation**				
No	1.00 [reference]		1.00 [reference]	
Yes	1.72 [1.40–2.13]	< 0.001	1.12 [0.97, 1.30]	0.135
**Birth weight**				
Normal (≥2.5 kg)	1.00 [reference]		1.00 [reference]	
Low birth weight (1.5 to < 2.5 kg)	1.56 [0.76–3.20]	0.231	1.30 [0.71, 2.39]	0.397
Very low birth weight (1.0 to < 1.5 kg)	3.90 [1.91–7.96]	< 0.001	2.27 [1.22, 4.23]	0.010
Extremely low birth weight (< 1.0 kg)	9.15 [4.47–18.71]	< 0.001	3.15 [1.68, 5.88]	< 0.001
**Congenital anomalies**				
No	1.00 [reference]		1.00 [reference]	
Yes	1.83 [1.18–2.84]	0.007	2.18 [1.52, 3.12]	< 0.001
**Completed gestational weeks at birth**				
Late/Moderate (32 to < 37 weeks)	1.00 [reference]		1.00 [reference]	
Very preterm (28 to < 32 weeks)	2.33 [1.79, 3.02]	< 0.001	1.41 [1.11, 1.79]	0.005
Extreme preterm (< 28 weeks)	4.84 [3.75, 6.24]	< 0.001	1.87 [1.43, 2.46]	< 0.001

uRRR, Unadjusted relative risk ratio; aRRR, adjusted relative risk ratio, CI; confidence interval.

Compared to those with normal weight at birth, the risk of death at discharge was significantly higher among those with very low birth weight (aRRR: 2.27, 95% CI: [1.22–4.23], *p* = 0.010) and those with extreme low birth weight (aRRR: 3.15, 95% CI: 1.68–5.88], *p* < 0.001).

The risk of death at discharge in admitted preterm newborns with congenital anomalies was over twice the risk in preterm with no congenital anomaly (aRR: 2.18, 95% CI: [1.52–3.12], *p* < 0.001). Compared to late/moderate preterm newborns, the adjusted risk of death was significantly higher among the very preterm newborns (aRR: 1.41, 95% CI: [1.11–1.79], *p* = 0.005) and the extreme preterm newborns (aRR: 1.87, 95% CI: [1.43–2.46], *p* < 0.001) (Table 3).

## Discussion

Three out of every ten preterm newborns (29.5%) admitted to the NICU died before discharge. The risk of death among these preterm newborns decreases as their gestational age increases. Those who were born extremely preterm (< 32 weeks gestation), had a higher risk of death compared to those born late preterm. Risk of mortality was higher among preterm new-borns with birth weight below 1.5 kg. The risk of death before discharge among preterm babies admitted at NICU was higher for babies with neonatal co-morbidities such as Haemorrhagic /haematological disorders of foetus, foetus/new-born infections, Respiratory disorders, Fetal growth disorders. The existence of complications and congenital anomalies at birth among preterm newborns increased the risk of death. The study, however, found that maternal conditions were not significant risk factors of death at the NICU.

Preterm birth remains a major cause of perinatal, neonatal, and infant mortality (3, 18, 24). The current study found that preterm birth accounted for about one-third of the deaths recorded over a two and quarter year period (January, 2017–April, 2019) at just one facility. A similar prevalence (26%) was reported by an earlier study that assessed neonatal deaths from 2003 to 2009 in northern Ghana (25). Even higher prevalence rates were recorded in South-East Nigeria (46.1%) for the period 2009 to 2013 (8) and 67.7% in South Africa (26). While significant progress has been made in reducing infection-related deaths among children generally, successes have been slow among neonates and more especially among preterms (4). In 2017, Ghana the Maternal Health Survey using a nationally representative sample, estimated neonatal mortality for the 5-year period preceding the survey as 25 deaths per 1,000 live births. The report indicated a very high uptake (98%) of antenatal care (ANC) from a skilled provider among pregnant women with about four out of five of them having four or more ANC visits (27). However, the uptake of ANC must be complemented with quality of care to mitigate neonatal deaths. Adu-Bonsaffoh et al. (14), recently reported that poor antenatal care among other factors predicted the incidence of preterm delivery in the same facility used for this study (28). Moreover, factors that predict preterm birth evidently account for a large proportion of preterm mortality (18). Also a cause for concern, is the preterm births that could have been avoided in low and middle-income countries (LMIC) as it has been found that about 14.2% of provider-initiated preterm birth in LIMC were not medically indicated (15).

Neonatal mortality has long been known to be associated with gestational age, with decreasing mortality in infants as gestational weeks approach full-term (10, 13, 29–31). Findings from this study corroborate these earlier reports because the risk of death among the preterm newborns decreased with increasing gestational age, with those extremely preterm having a higher risk of death compared to those born late preterm. D'Onofrio et al. (28), reported similar findings in their study that extreme preterm birth with a gestational period of 23–27 weeks was a predictor of infant mortalityClick or tap here to enter text.. Jacob et al. (2015), also found that mortality at lower gestational ages was most commonly attributed to extreme preterm birth with the attendant complications (32). About three out of four babies born at 24 weeks of gestation do not survive, one out of three babies die at 25 weeks of gestation and one out of four babies dies at 26 weeks of gestation before discharge from the hospital (33). The risk of death among these preterm newborns significantly increased with the presence of congenital anomalies and complications including hemorrhagic/hematological disorders of the fetus, respiratory disorders, and fetal growth disorders. Preterm birth complications is one of the three leading causes of death worldwide (3). Hyperglycemia occurring on the first day of life in extremely preterm infants (34), increased severity of respiratory failure (35), necrotizing enterocolitis, intraventricular hemorrhage in extreme to very preterm population (32, 36, 37), and birth asphyxia (25) have also been implicated as predictors of preterm deaths. Fetal and newborn infections however, did not siginificantly increase the risk of mortality. This supports earlier observations of significant reductions in infection-related deaths among children (4). These findings do not however, eliminate infection from the causes of preterm deaths as some studies have found its association with the latter (19, 36, 37).

Birth weight was also negatively associated with death in NICU. A very low birth weight (1.0 to < 1.5 kg) and extremely low birth weight (< 1.0 kg) significantly increased the risk of preterm death. Earlier findings have also indicated low birth weight (LBW) as a predictor of death in the first 24 hours after birth (38). Extremely low birth weight is significantly associated with death in the first 6 days after birth (7) and neonatal death before discharge from the health facility (26). A meta analysis of empirical data from 12 randomized controlled trials conducted in 10 low-to-middle-income countries revealed that maternal supplementation with multiple micronutrients (a minimum of 1 recommended dietary allowance of multiple micronutrients), compared to iron–folic acid (IFA) supplementation alone during pregnancy, increased birthweight thereby, protecting one in ten infants from LBW (39). Maternal micronutrient supplementation (MMS) also reduces fetal growth restriction, prematurity, and neonatal mortality (40, 41). In Ghana, it has also been found that MMS reduces the risk of LBW compared to IFA supplementation alone (42). Moreover, the WHO has also indicated that there is high-certainty evidence that MMS reduces the risk of having LBW neonates compared with IFA supplements only, which is the standard care. However, MMS barely improved other fetal and neonatal outcomes including preterm birth rates. Hence, WHO has not yet recommended MMS for pregnant women. More research is required to ascertain if MMS will improve other neonatal outcomes, and how the micronutirents can be best combined into a single supplement (43). Furthermore, careful consideration must be given to the cultural setting as the economic situation, cultural representations of motherhood, and the unpredictable demands of the pregnant body could influence pregnancy food practices (44).

While birth weight is highly correlated with gestational age, studies that controlled for body size have reported a consistent relationship between prematurity and increased risk of morbidity and mortality (45). More robust survival predictive models based on a combination of gestational age and birth weight still predicts poorer outcomes for preterms classified as small for gestational age (SGA) compared to normal weight or term births (46).

Kangaroo mother care (KMC) is another intervention is being explored to improve the survival of low birthweight and preterm infants though the uptake is slow in low-to-middle-income countries (47–49). There is evidence of its integration into healthcare facilities providing newborn care for LBW in Ghana with designated wards in some hospitals (50, 51). This intervention is increasingly being implemented/scaled-up in different parts of the country.

Maternal factors and delivery conditions were not significant risk factors of preterm death before discharge from NICU in the current study. This supports the earlier findings of Iyoke et al. that survival was not dependent on maternal risk factors in a retrospective review of singleton preterm and term births from 2009 to 2013 in a teaching hospital in Nigeria (52). Contrary findings have also been reported by Bayou and Berhan, who found that obstructed labor, malpresentation, antepartum hemmorrhage and hypertensive disorders of pregnancy were significant predictors of high perinatal mortality (53). Additionally, Adu-Bonsaffoh et al., indicated that maternal age, hypertensive disorders and preterm rupture of membranes were associated with preterm birth in the same facility the current study was conducted (14). The limited data from the records reviewed in this study did not allow for the effect of maternal lifestyle factors including smoking and alcohol intake to be assessed although they have been implicated in literature as influencing preterm births and some negative neonatal outcomes (54–56).

There is the need for further research into interventions that are safe, effective, and scalable in limited-resource settings, where most preterm-associated infant mortality occurs (5). It might be useful to focus on hospital care related interventions delivered during labour and birth, which have been found to be most effective in reducing neonatal deaths. The KMC and the use of antenatal corticosteroids can avert about half and one third of preterm-related deaths, respectively (57, 58). Also, low and middle-income countries should improve their availability and quality of data on preterm births as recommended by the WHO in the framework of the Global Strategy for Women's, Children's and Adolescents' Health (59, 60). Currently, most of the data available are from facility-based research studies, which were often conducted in tertiary facilities and are largely not nationally representative (5) as is the case for this study. Efforts in this post-2015 era should advance beyond just promoting child survival to reducing child morbidity and ensuring healthy development. It is crucial that as child survival improves, children are not left with impairments (3).

### Study limitations

Although the study design adequately answered the study objective, the retrospective record review employed did not allow for some relevant clinical indicators of preterm birth implicated in literature, such as maternal lifestyle factors including smoking and alcohol intake, medications used by mothers during pregnancy, and record of antenatal care among others to be assessed. For the same reason, a very important predictor of preterm outcome such as single/multiple birth was not extracted. Furthermore, authors are unable to report on other method of determining gestational age and its possible impact on preterm classification.the data used in this study was obtained from just one tertiary facility. Hence the sample is not nationally representative and findings cannot be generalized for the country.Since the data was extracted from hospital records, issues of missingness of information on specific characteristics was common. This was evident from the flow chart in Figure 1 where 71 observation did not have data on key variables. Also, the method of measurement of information such as gestational age at birth may vary from one record to the other. Also, the issue of complete omission of information of some records cannot be ignored given that past records of hard copies spanning over a 3 years were reviewed.

## Conclusion

This study demonstrate that maternal factors are not significant risk factors of preterm deaths. Gestational age, birth weight, presence of complications and congenital anomalies at birth are significantly associated with preterm deaths. Interventions delivered during labour and birth including kangaroo mother care and the use of antenatal corticosteroids should be scaled up to ensure nationwide coverage so as to reduce preterm deaths. Additionally, further research involving national level data on preterm deaths and associated causes is recommended.

## Data availability statement

The original contributions presented in the study are included in the article/supplementary material, further inquiries can be directed to the corresponding author.

## Ethics statement

The studies involving human participants were reviewed and approved by Korle Bu Teaching Hospital, Institutional Review Board, IRB No: KBTH-IRB/00010/2019. Written informed consent from the participants' legal guardian/next of kin was not required to participate in this study in accordance with the national legislation and the institutional requirements.

## Author contributions

HT conceived the study, prepared the data collection guide, and collected the data. HT and DOA researched the literature. SKA and YA were involved in the data analysis and result interpretations. SKA wrote the first draft of the manuscript. All authors reviewed, proofread, and approved the final version of the manuscript.
